# Identification of a discriminative metabolomic fingerprint of potential clinical relevance in saliva of patients with periodontitis using ^1^H nuclear magnetic resonance (NMR) spectroscopy

**DOI:** 10.1371/journal.pone.0182767

**Published:** 2017-08-24

**Authors:** Matthias Rzeznik, Mohamed Nawfal Triba, Pierre Levy, Sébastien Jungo, Eliot Botosoa, Boris Duchemann, Laurence Le Moyec, Jean-François Bernaudin, Philippe Savarin, Dominique Guez

**Affiliations:** 1 Paris 13 University, Sorbonne Paris Cité, CSPBAT, UMR 7244, CNRS, Bobigny, France; 2 APHP, Department of Periodontology, Bretonneau Hospital, Paris-Descartes University, Paris, France; 3 APHP, Department of Public Health, Tenon Hospital, Paris, France; 4 UMR-S1136 (EPAR team), INSERM UPMC, Sorbonne Universités, Paris, France; 5 APHP, Department of Pneumology, Avicenne Hospital, Bobigny, France; 6 UBIAE, Univ Evry, Université Paris-Saclay, Evry, France; 7 UPMC Paris 6, Sorbonne Universités, Paris, France; 8 Paris 13 University, Sorbonne Paris Cité, EA2363, Bobigny, France; University of Texas at Austin, UNITED STATES

## Abstract

Periodontitis is characterized by the loss of the supporting tissues of the teeth in an inflammatory-infectious context. The diagnosis relies on clinical and X-ray examination. Unfortunately, clinical signs of tissue destruction occur late in the disease progression. Therefore, it is mandatory to identify reliable biomarkers to facilitate a better and earlier management of this disease. To this end, saliva represents a promising fluid for identification of biomarkers as metabolomic fingerprints. The present study used high-resolution ^1^H-nuclear magnetic resonance (NMR) spectroscopy coupled with multivariate statistical analysis to identify the metabolic signature of active periodontitis. The metabolome of stimulated saliva of 26 patients with generalized periodontitis (18 chronic and 8 aggressive) was compared to that of 25 healthy controls. Principal Components Analysis (PCA), performed with clinical variables, indicated that the patient population was homogeneous, demonstrating a strong correlation between the clinical and the radiological variables used to assess the loss of periodontal tissues and criteria of active disease. Orthogonal Projection to Latent Structure (OPLS) analysis showed that patients with periodontitis can be discriminated from controls on the basis of metabolite concentrations in saliva with satisfactory explained variance (R2X = 0.81 and R2Y = 0.61) and predictability (Q2Y = 0.49, CV-AUROC = 0.94). Interestingly, this discrimination was irrespective of the type of generalized periodontitis, *i*.*e*. chronic or aggressive. Among the main discriminating metabolites were short chain fatty acids as butyrate, observed in higher concentrations, and lactate, γ-amino-butyrate, methanol, and threonine observed in lower concentrations in periodontitis. The association of lactate, GABA, and butyrate to generate an aggregated variable reached the best positive predictive value for diagnosis of periodontitis. In conclusion, this pilot study showed that 1H-NMR spectroscopy analysis of saliva could differentiate patients with periodontitis from controls. Therefore, this simple, robust, non-invasive method, may offer a significant help for early diagnosis and follow-up of periodontitis.

## Introduction

Periodontitis is characterized by the loss of the supporting tissues of the teeth in an inflammatory-infectious disease context [1–4]. The prevalence of periodontal disease remains high [5, 6]. Periodontitis is often diagnosed late, with important tissue damage already present and, if untreated, eventually leads to loss of teeth. Therefore, the main objective of periodontal treatment is to control infection in a sustainable manner and, secondarily, to repair damaged tissues. However, restoration of a “well-balanced host-microbial symbiotic state” [7–10] in the oral cavity at the level of periodontal structures may be difficult. Moreover, regeneration of diseased periodontal tissues by surgical or non-surgical techniques still remains hypothetical. In addition, while the replacement of lost tooth roots by dental implants can compensate for dental disability, high-risk patients for periodontal disease are also high-risk patients for implant therapy failure [11]. Finally, periodontitis may be associated with systemic diseases such as diabetes or cardiovascular disease [12, 13]. For all these reasons, the detection of early stage disease of periodontitis is essential. Currently, diagnosis relies on clinical and X-ray examination. Unfortunately, clinical signs of tissue destruction occur late, and clinical monitoring is time-consuming, subject to measurement error, and often poorly tolerated by patients. Therefore, it may be hypothesized that identifying precise and reliable biomarkers of disease activity would allow for better management of the disease.

Human saliva *per se* is a complex biological fluid secreted by major and minor salivary glands. In addition to true saliva components, oral fluid contains a wide spectrum of *compounds* originating from multiple local and systemic sources as a complex microbiota, serum and tissue components carried by the gingival crevicular fluid (GCF). [14–17]. GCF flows into the oral cavity from periodontal pockets and the intensity of this exudate, whose composition is close to that of serum, varies as a function of gingival inflammation [18]. In the present report, the term saliva is used to designate the collected oral fluid including all its components.

“Omics” methods are promising techniques in the field of system biology allowing a global evaluation of the metabolic changes in a biologic milieu. Indeed, if various reports dealt with “omics” in the saliva, they were mostly proteomic studies and less often metabolomic studies. Concerning periodontitis, it has been shown that “omics” technologies, such as proteomics, are particularly promising for periodontitis research [3, 19–25]. Recently, using a method based on gas chromatography and mass spectrometry on a set of 19 volunteers, Kuboniwa et al. showed that metabolic analysis of saliva in periodontitis may be of potential clinical interest [25]. Proton nuclear magnetic resonance (^1^H NMR) spectroscopy analysis is considered as a robust, reproducible quantitative method having the ability to identify a maximal number, both hydrophilic and lipophilic metabolites via their unique spectral patterns referred as a metabolomics signature [26]. This method has been used to explore various physiological fluids in pathological conditions [27–29]. However, there have been only a few reports on ^1^H NMR spectroscopy investigations of saliva, almost exclusively for general diseases or physiological studies [30–35]. Indeed reports concerning periodontitis are extremely rare [25, 36, 37]. Therefore, we designed the present pilot study using ^1^H NMR spectroscopy of saliva with the goal of determining its potential clinical relevance through the identification of a metabolomics signature of active periodontitis in patients compared to healthy controls matched by age, gender, and smoking habits.

## Material and methods

### Ethics statement

The protocol was approved, under n°10–047, by the medical ethics international review board of Paris North Hospitals (N°IRB00006477) in accordance with the guidelines for the protection of human subjects. Written informed consent was obtained from all patients and controls prior to their participation.

### Study population and design

Twenty-six patients (10 men and 16 women) with chronic and aggressive generalized periodontitis (18 and 8 respectively) were recruited from among the outpatients at the Department of Periodontology of Bretonneau Hospital in Paris, France (AP-HP, University Paris Descartes, Paris, France). The diagnostic criteria for chronic and aggressive generalized periodontitis were defined according to the classification proposed at the International Workshop for the Classification of Periodontal Diseases and Conditions in 1999 [38]. Healthy controls (n = 26) were simultaneously recruited from dental students or medical staff and were matched by age (± 5 years), gender, and smoking habits. Both patient and control groups were balanced for ethnicity and socio-economic levels.

#### Inclusion criteria

The common inclusion criteria for patients with periodontitis or healthy controls were: (i) age range of 18–64 years (ii) health insurance benefit (iii) no systemic diseases, (iv) no antibiotics taken during the past three months, (v) no regular alcohol consumption and (vi) women not pregnant or nursing. Furthermore, for patients no initial therapy was applied within the 6 months before the study. After recruitment, patients and healthy controls who agreed to participate were asked to complete a questionnaire including the following sections: demographic and socio-economic data, medical history, dental habits and dental care utilization, and tobacco consumption. Smoking was quantified in pack-years, and patients’ smoking habits were linearly stratified into 5 ordered categories [39, 40].

#### Definition of periodontitis

Periodontitis is defined as an active destruction of the periodontal tissues. The diagnosis was based on clinical and radiographic assessment: clinical measures of Pocket Depth (PD) and Clinical Attachment Loss (CAL), and evaluation of alveolar bone loss by X-ray [41, 42]. Moreover, a Bleeding On Probing (BOP) was included to characterize the degree of gingival inflammation, active periodontitis being not reflected only by attachment or bone loss measurements [43]. Periodontitis cases were defined according to CDC-AAP (Centers for Disease Control—American Academy of Periodontology) definitions updated in 2012 [42]. Observation of at least ≥ 2 interproximal sites with CAL ≥ 3 mm, and ≥ 2 interproximal sites with PD ≥ 4 mm (not on the same tooth) or one site with PD ≥ 5 mm was necessary to diagnosis. Periodontitis was diagnosed as localized if ≤30% and generalized if >30% of the sites were affected [38].

According to the classification proposed at the International Workshop for the Classification of Periodontal Diseases and Conditions in 1999 [38], Chronic Periodontitis (CP) resulting from poor oral hygiene with the presence of bacterial plaque is prevalent in most adults. The rate of progression of attachment and bone loss is slow to moderate. In contrast, aggressive periodontitis (AP) is characterized by a rapid attachment loss and alveolar bone destruction in patients associated with varying degrees of bacterial plaque.

#### Clinical data assessment

The following parameters were appraised: Number of Residual Teeth (NRT) and Decay Missing Filled (DMF) for dental history; Plaque Control Record (PCR) [44] and Gingival Bleeding Index (GBI) [45] for presence of bacterial plaque and gingival inflammation. For patients with periodontitis, in a second clinical session after dental prophylaxis, indices assessing the severity and extent of the periodontitis i.e., PD, and CAL were recorded. Simultaneously, BOP was scored [46, 47]. Clinical parameters were assessed at four sites on each tooth (mesiobuccal, distobuccal, mesiolingual, and distolingual) using a manual periodontal probe (Hu-Friedy, Chicago, Il, USA), at 20 g of pressure. Tooth sites excluded from the examination were impacted teeth, retained roots, and teeth with indeterminable cemento-enamel junction. The third molar was excluded from analysis. For each site, CAL was distributed into groups (mild CAL: 3–4 mm, moderate CAL: 5–6 mm, and severe CAL: ≥ 6 mm) and their percentage was assessed for each patient. The highest value of tissue destruction was determined as CALMAX and the average CAL (CALMEAN) as well as the mean PD (MPD) were computed. The assessment of bone loss (BL) was performed on periapical radiographs of patients. Radiographs were taken using a standardized long-cone paralleling technique. Bone loss was measured according to the modified classification of Hugoson & Jordan [48]. For this study, a professional dentist trained for the evaluation and sampling procedure performed all clinical assessments and collections of saliva.

#### Collection of stimulated saliva

The collection of saliva was done under standardized conditions, between 09:00 and 11:00 am after paraffin wax-stimulation and to prevent blood contamination, before the assessment of clinical parameters. A volume of 10 mL saliva was collected (±5 min), the pH was recorded and the sample immediately stored at -25°C. Patients and controls were required to refrain from eating, drinking, chewing gum, and tooth brushing for at least 2h prior to the sample collection.

### ^1^H NMR spectroscopy

For NMR analysis, samples were thawed at room temperature and centrifuged for 2 min at 2000g before analysis. A volume of 0.6 mL of saliva was placed into a 5-mm-diameter specific tube together with 0.1 mL of D_2_O. The proton spectra were acquired at 500 MHz on a Varian Unity Inova^®^ spectrometer at 25°C. A signal was acquired after a 90° pulse of 32K data points on a spectral window of 5000 Hz. The relaxation delay was 4s. The water signal was suppressed by a pre-saturation sequence using low-power irradiation (0.03 W for 2s) on the water-signal frequency during the relaxation delay. The resulting free induction decays obtained with 128 transients were processed by NMR Pipe software. A Fourier transformation was applied with an exponential window function to produce a 1 Hz broadening line. Spectra were phased and a multipoint linear baseline correction was applied. Each spectrum was referenced using a propionic acid signal (1.04 ppm). The spectral region between 0–9 ppm was divided into 9000 spectral regions of 0.001 ppm width, called buckets, using in-house C code. The water region was excluded [5.1; 4.3 ppm]. The icoshift tool for Matlab (Matlab^®^ 2014, The Mathworks Inc., Natick, MA, USA) was used for realignment [49]. Each bucket was integrated and scaled using probabilistic quotient normalization [50].

### Statistical analysis

#### Multivariate analysis

Principal component analysis (PCA) was performed on clinical and NMR data. PCA was used to assess the homogeneity of the cohort and to detect and exclude any outliers defined as observations located outside the 95% confidence region of the model. For clinical data analysis, PCA was performed on the patient group to assess the homogeneity of the patient cohort regarding the clinical data and to detect any relationships between clinical variables. This analysis was performed with the SIMCA-P statistical package (Umetrics, Umeå Sweden). For NMR data analysis, PCA was performed with an in-house Matlab^®^ code using the same algorithm as SIMCA-P to detect any group separation based on signal variability.

An orthogonal projection to latent-structure (OPLS) analysis was run to discriminate patients and controls. Compared to the classical projection of latent-structure analysis (PLS), this method improves interpretation of the spectroscopic variations between discriminated groups by removing the orthogonal information that had no impact on the discrimination.

The goodness-of-fit parameters of the OPLS model R2Y and Q2Y were calculated. R2Y represents the explained variation of the Y matrix. Q2Y was calculated with the K-fold (K = 7) method and was used to estimate the predictability of the model. R2Y = 1 indicates a perfect description of the data by the model while Q2Y = 1 indicates a perfect prediction of new data. Score and loadings plots are used to illustrate the results. Each point in the score plot represents the projection of an NMR spectrum (and thus a control or patient sample) on the predictive (horizontal axis) and orthogonal components of the model (vertical axis). The loadings plot represents the covariance between the Y-response matrix and the signal intensity of the various spectral domains. Colours were also used in the loadings plot depending on correlations between the corresponding bucket intensity and the Y variable. Positive signals correspond to those metabolites that had an increased concentration in patients. Conversely, negative signals correspond to those metabolites that had an increased concentration in controls.

An internal validation of the OPLS model was performed using a permutation test (999 random permutations of group membership). The aim of this test was to evaluate whether our OPLS model, built with groups, were significantly better than any other OPLS model obtained by randomly permuting the original group attributions. The model is validated when R2 and Q2 are higher for the non-permutated compared to those of all permutated models.

To estimate the ability of the model to correctly classify a new data set, a receiver operating curve (ROC) was built using the predictions of each cross-validation set and an area under this curve was calculated (CV-AUROC).

#### Multivariate logistic regression

To study the risk associated with periodontitis (dependent variable), a descending stepwise multivariate logistic regression was performed. Prior to the logistic regression, a univariate analysis by a Mann—Whitney test was used to make the selection of the metabolites that will be computed for logistic regression analysis. Variables with *P *≤ 0.2 on univariate analysis were included in the stepwise logistic regression analysis to compare patients to controls. For the multivariate phase, *P* < 0.05 was considered statistically significant. [51].

## Results

A total of 52 subjects, 26 patients with generalized periodontitis (18 CP and 8 AP) and 26 controls were recruited. One control was not considered because of doubts concerning his periodontal health. A total of 25 controls were retained, one control being matched with 2 different cases.

Demographic characteristics of controls and patients and the clinical parameters of the patient group are summarized in Table 1. Clinical parameters were irrelevant for controls as they were healthy subjects free of any periodontitis.

**Table 1:** Clinical data of patients with periodontitis and controls.

		periodontitis	controls
N^1^		26	25
	men	10	9
	women	16	16
Age	years (mean ± SD)	42.4 ± 12.8	40.7±12.4
Smoking habits^1^	none	13	12
	former	2	2
	current	11	11
NRT^2^ (mean ± SD)		26 ± 2.4	26.6 ± 2.1
Generalized periodontitis^3^	chronic	18	0
	aggressive	8	
Severity index ^4^	mild	1	/
	moderate	6	
	severe	19	
Mean DMF^5^		8.23	/
Mean affected sites (%)		48.5	/
Mean PCR^6^ (%)		61.2	/
Mean BOP^7^ (%)		35.0	/
PD^8^ (mean ± SD, in mm)		3.8± 0.5	/
CAL^9^ (mean ± SD, in mm)		4.1± 0.8	/
BL^10^	Grade 1	5	/
	Grade 2	12	
	Grade 3	9	

^1^smoking habits were stratified according to [39].

^2^NRT: number of residual teeth;

^3^controls were free of periodontitis;

^4^according to [42];

^5^DMF: decay missing filled;

^6^PCR:plaque control record;

^7^BOP:bleeding on probing;

^8^ PD: pocket depth;

^9^CAL:clinical attachment loss;

^10^BL:bone loss [grade according to [48]

The number of remaining teeth was similar between patients and controls (26 ± 2.4 vs 26.6 ± 2.13, NS). For patients, MPD and attachment loss (CAL) were 3.82 ± 0.5 mm and 4.12 ± 0.78 mm, respectively.

### Multivariate analysis of clinical data

**Fig 1:**
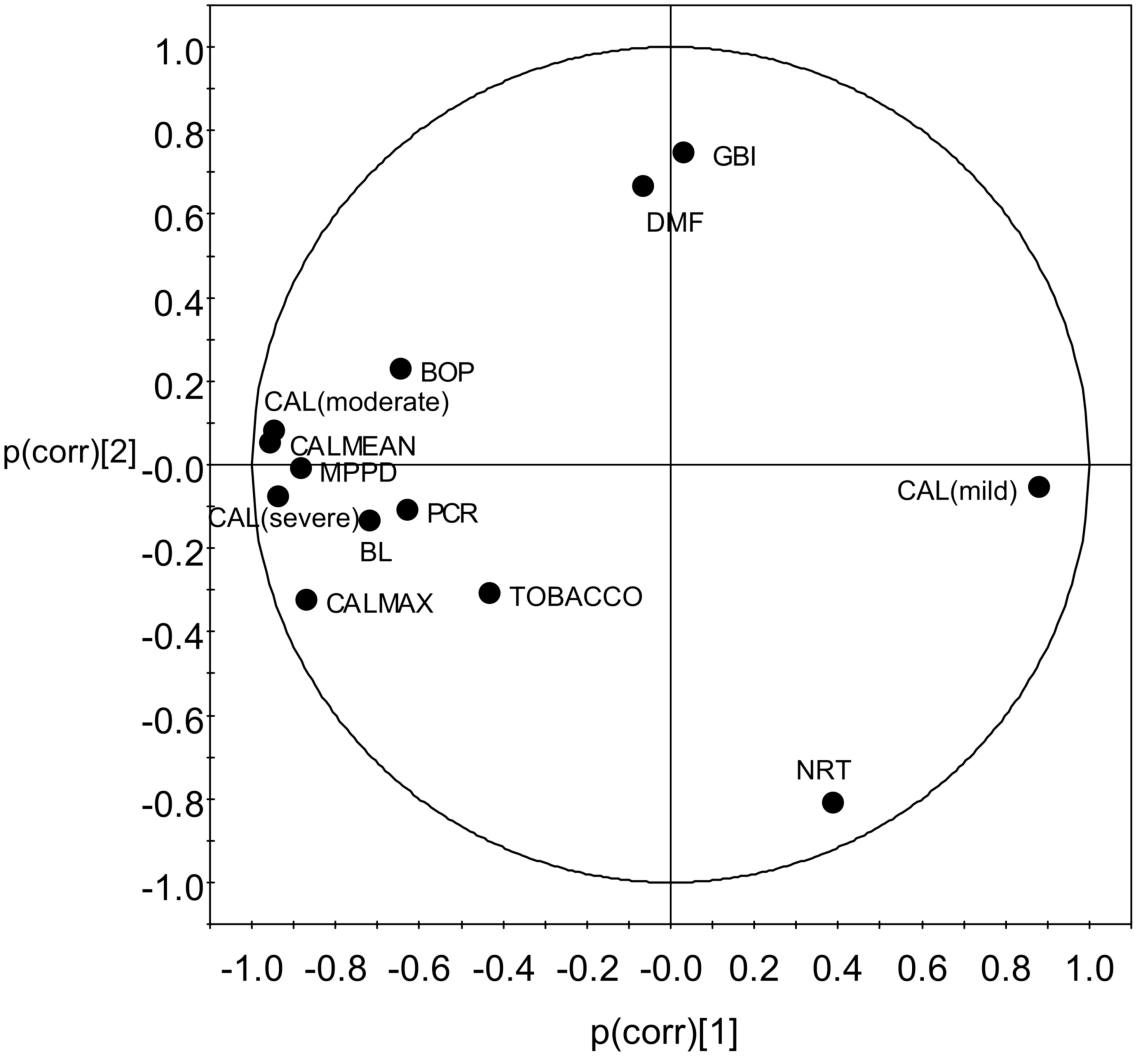
Loadings plot of the principal component analysis (PCA) performed on clinical variables of periodontitis. Loadings are scaled so that the correlated variables correctly explained by the components are found close together and near the correlation circle. BL: bone loss; BOP: bleeding on probing; CAL: clinical attachment loss, expressed as a mean (CALMEAN) or according to the severity of the loss (CALmild, CALmoderate, and CALMAX); DMF: decay missing filled; MPPD: mean pocket depth; NRT: number of residual teeth; PCR: plaque control record; TOBACCO: smoking habits (for details see Materials and methods).

PCA was performed on periodontal variables from the 26 patients. The score plot is shown in Fig 1.

No outlier was detected in the plan defined by the first and second principal components, PC1 and PC2, which correspond to 63% of the variability in the clinical data. Considering the positions of variables on the loadings plot, the loss of periodontal tissues (including CALMEAN, percentage of sites with severe or moderate CAL, BL), the criteria of active disease (MPD), and the percentage of bleeding sites (BOP) were strongly correlated and contribute strongly to PC1 variability.

### Multivariate analysis of metabolomics data

**Fig 2:**
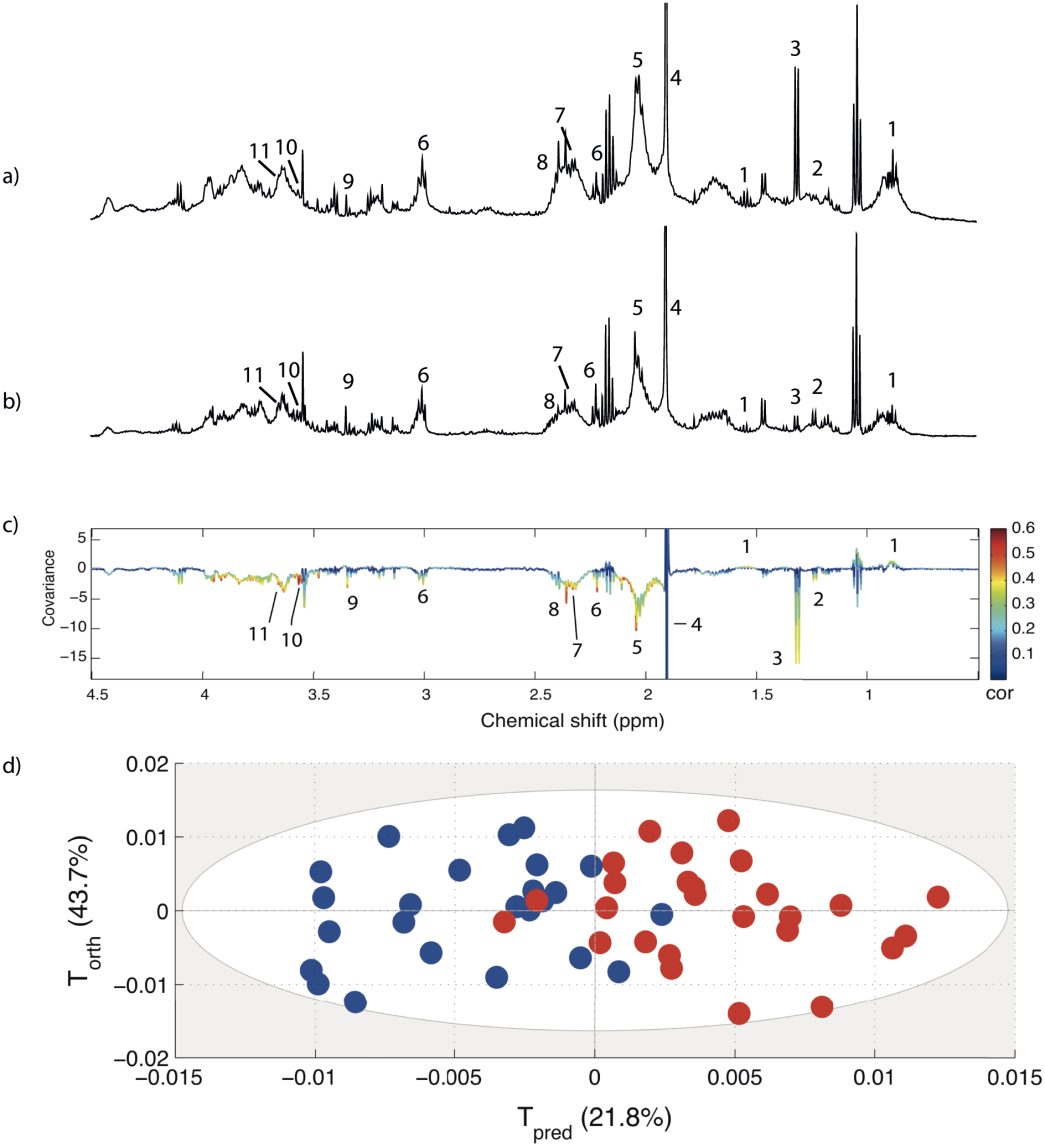
Examples of NMR spectra in saliva and OPLS metabolomic analysis. a,b) Representative ^1^H-NMR spectra obtained in (a) a control individual and (b) a case individual. c) Orthogonal projection to latent structures (OPLS model) of ^1^H-NMR spectra obtained in saliva from periodontitis patients (red dots) and healthy controls (blue dots) according to the predictive (Tpred) and not predictive (Torth) components obtained from the OPLS model. d) OPLS loadings plot showing the discriminant metabolites between patients with periodontitis and controls. Variations of metabolites are represented using a line plot between 0–9 ppm. Positive signals correspond to metabolites present at increased concentrations in the patient group. Negative signals correspond to metabolites present at increased concentrations in the control group. The buckets are labelled according to metabolite assignment (1. butyrate; 2. fucose; 3. lactate; 4. acetate; 5. N-acetyl of glycoprotein; 6. GABA; 7. 3-hydroxybutyrate; 8. pyruvate; 9. methanol; 10. threonine; 11. ethanol).

Two examples of NMR spectra obtained in saliva from a control individual and a patient case are respectively shown on Fig 2a and 2b.

OPLS analysis was performed and showed a good separation between controls ^1^H NMR spectra and those of patients (Fig 2c). The quality parameters of the model were R2Y = 0.57 and Q2Y = 0.48.

In this pilot study, metabolomics profiles, interestingly, did not allow us to separate generalized AP from generalized CP as R2, Q2 and CV-AUC were respectively 0.09, -0.62 and 0.2 for one component.

The ability of this model to classify control and patient samples was confirmed by the cross-validation area under the ROC curve (CV-AUROC) of 0.91.

### Identification of metabolites

By analysing the loadings plot, the main metabolites responsible for the discrimination were identified (Fig 2d). Compared to controls, higher concentrations of butyrate (short chain fatty acids) were observed in patients. Conversely, lower concentrations of fucose, lactate, acetate, N-acetyl, gamma-aminobutyrate (GABA), 3-D-hydroxybutyrate, pyruvate, methanol, threonine, ethanol were observed in patients (Table 2) in which the peak number corresponds to the spectral assessment shown in Fig 2d.

**Table 2:** Main metabolites identified to discriminate periodontitis from controls according to the loadings plot analysis. Correlation between bucket intensities and discriminated group members are given with the associated p value.

		Chemical shift (ppm)	Correlation coefficient	p value
1	Butyrate	0.89 and 1.54	0.43	0.001637
2	Fucose	1.23	-0.38	0.005951
3	Lactate	1.31	-0.37	0.007531
4	Acetate	1.91	-0.46	0.000683
5	N-acetyl of glycoprotein	2.04	-0.51	0.000132
6	GABA	2.22 and 3.0	-0.49	0.000263
7	3-Hydroxybutyrate	2.33	-0.49	0.000263
8	Pyruvate	2.36	-0.5	0.000187
9	Methanol	3.35	-0.44	0.001234
10	Threonine	3.56	-0.57	1.3E-05
11	Ethanol	1.20 and 3.65	-0.3 and -0.49	0.032448 and 0.000263

### Logistic regression

A univariate analysis was performed as a preliminary step to select the metabolites to be computed for the stepwise regression analysis. Prior to the univariate analysis a binarization of the variables was made by comparison of their respective histograms. Due to the large number of significant variables with regards to the total number of patients (26) and controls (25), we kept the five variables (lactate, GABA, butyrate, threonine, hydroxybutyrate) with *P *≤ 0.2 (S1 Table in supplementary material). and already identified in the OPLS loadings plot analysis (Table 2) as potentially clinically relevant. Table 3 reports the stepwise logistic regression analysis: 5 metabolites were entered in the model and 3 metabolites (lactate, GABA and butyrate) were recognized as the main potential independent biomarkers for periodontitis diagnosis. The best positive and negative predictive values were those resulted from the computing of the positive and negative predictive values corresponding to all the associations of the values of the three variables of the final table of the multivariate logistic regression: the group lactate = 1-GABA = 1-butyrate = 1 emerged (PPV 0.77) (Table 4).

**Table 3:** Multivariate logistic regression analysis.

Variables	p	OR	95%CI
*Initial table* ^1^			
Lactate	0.0115	13.672	(0.001–44.622)
GABA	0.0328	15.247	(1.249–186.203)
Butyrate	0.1094	7.775	(0.631–95.796)
Threonine	0.1690	1.542 E-134	(0–7.703E56)
Hydroxybutyrate	0.2421	2.67E-14	(4.816E-37–1481137133.037)
*Final table*^2^			
Lactate	0.0088	13.464	(1.924–94.208)
GABA	0.0043	33.480	(2.999–373.813)
Butyrate	0.0291	14.816	(1.316–166.833)

^1^Analysis of the 5 variables chosen according to the preliminary univariate analysis (see text and supplementary material; analysis done on 51 samples; R^2^ = 0.394);

^2^ from the final results of the logistic regression 3 variables were independently associated to periodontal disease (analysis done on the 51 samples; R^2^ = 0.355).

OR: Odds ratio; CI: confidence interval; GABA: gamma-aminobutyrate.

**Table 4:** Evaluation of the clinical significance of the grouped lactate-GABA-butyrate considered as one.

	PPV	NPV	Se	Sp
Value (95% CI)	0.77 [0.65–0.88]	0.86 [0.76–0.95]	0.89 [0.80–0.97]	0.72 [0.06–0.84]
sigma	0.06	0.05	0.04	0.06

PPV: positive predictive value; NPV: negative predictive value; Se: sensitivity; Sp: specificity.

## Discussion

^1^H NMR spectroscopy is a reliable, cost-effective method for analysis of biofluids [29, 34]. Only a few studies on saliva using this method have been reported, mostly in general diseases, for physiological studies, or for illicit drug uptake [32, 36, 52]. Our group has already investigated human saliva in the context of sarcoidosis [34]. It also must be noted that it has been recently reported that investigating saliva is less influenced by diet than urine [35]. Therefore, this method can be considered as a method of choice for investigating periodontal disease. In the present pilot study, using ^1^H NMR spectroscopy, we carried out the metabolic profiling of saliva collected in periodontitis patients before any treatment, and in healthy controls matched for age, gender, and tobacco status.

### Representation of the periodontitis population by this cohort of patients

Clinical data indicated that our sample of 26 patients, aged from 18 to 64, was representative of patients with active periodontitis according to the usual criteria (*i*.*e*. inflammation, PD, CAL and BL). Cases were recruited according to the periodontal criteria (see Materials and methods) eliminating stable disease or morphologic abnormalities without inflammatory process (*i*.*e*. recession).

### Discrimination between controls and patients

Comparison of the ^1^H NMR spectra through multivariate statistical analyses showed significant differences between controls and patients in saliva metabolomic profiles. Because of the stringent selection criteria used for patients and controls that excluded most of the confusing bias, it can be considered that disease-specific content was observed in saliva metabolomic evaluations, illustrated by variations of the NMR signal intensity of eleven metabolites when comparing the 2 groups.

Interestingly, in this pilot study, the metabolomic profiles observed in generalized CP and AP were not distinct. Periodontitis, characterized by tissue destruction, is largely considered to be due to an inflammatory-infectious process. Consequently, the metabolomic fingerprints observed in saliva may be considered primarily to be related to tissue destruction mechanisms and, therefore, dependent on the severity of the disease rather than on the type of periodontitis. A second possible explanation suggests that the observed metabolite concentration changes may be related to the products of the pathogenic bacterial population. As the disease progresses, the sub-gingival environment is known to change with regard to oxygen tension, redox potential, pH, and availability of host-derived macromolecules [53–56]. These changes are responsible in a cause-and-effect way for modulation of the composition of the bacterial community [57]. Metabolomic profiles observed in patients with periodontitis may reflect a disease-associated microbiota, which may share similar metabolite profiles in generalized CP and AP, even if bacterial species found in these clinical entities may be different. It may be hypothesized that the source of the major identified metabolites in oral fluid, allowing us to distinguish patients from controls, is from bacterial populations colonizing the periodontal tissues.

### Metabolic changes in the oral fluid

A metabolomic profile associated with generalized periodontitis was clearly identified in the oral fluid. In particular, a significant increase of butyric acid was found. Some types of anaerobic periodontal bacteria such as *P*. *gingivalis* and *F*. *nucleatum*, belonging to *Bacteroidetes* and *Fusobacteria* phyla, produce this metabolite [58–60]. Butyrate, as well as other short chain acids (SCAs), is released into the microenvironment from infection sites and thought to contribute to the pathogenesis of periodontitis through impairment of defence cells or fibroblasts and in epithelial cell functions [58, 60, 61]. Thus, SCAs as butyric acid are found to be associated with periodontal inflammation [62]. In a longitudinal study of patients with chronic periodontitis receiving periodontal treatment, it was reported after chromatography analysis that the concentration of butyric acid in gingival crevicular fluid decreased to reach the same levels found in the healthy control group [62]. However, during the long-term observation period after therapy, gradually increasing concentrations of butyric and isovaleric acids seemed to be associated with recolonization of periodontal pathogens [62]. Pathogenic bacteria are known to be harboured in deep pockets where they find favourable conditions for their development [63]. Increased concentrations of butyrate may indicate a growth in pathogenic subgingival microorganisms linked to the progression of periodontal destruction. On the other hand, in our study, disease-specific increases of butyrate concentrations are associated with reduced levels of other SCAs such as lactic acid, acetic acid, formic acid, or γ-aminobutyric acid. The marked reduction of lactate in patients could reflect a shift in the microbial composition of commensal bacteria in the healthy oral cavity as well as in other mucosal ecological niches. This may include lactic acid bacteria [64], producers of lactic acid [65, 66]. In mucosal compartments, these organisms are known to contribute to the control of microbiota by competing with other microorganisms for adherence to epithelial cells and by producing antimicrobial compounds [65, 67]. This symbiotic relationship includes a complex molecular cross-talk between LAB and the host [68]. For instance LAB species can modulate immunological functions in the digestive tract, such as the enhancement of the ratio between anti-inflammatory (IL-10, TGFβ) and pro-inflammatory (IL-1β, IL-3, IL-4) cytokines [69]. On the other hand, the immune system selects the LAB species to be accepted. Therefore, vaginal microbiota of healthy reproductive women are often dominated by LAB species, contributing to the maintenance of good health by producing acid, hydrogen peroxide, and bacteriocin-like substances [65, 70]. Lactic acid is able to inhibit the growth of many bacteria, particularly Gram-negative species, by disrupting their outer membrane [71, 72]. With this ability to produce substantial amounts of lactic acid, oral LAB species might be recognized as beneficial species for the microenvironment between the teeth and the gum by inhibiting the growth and preventing the colonization of exogenous Gram negative pathogens [73], even if carbohydrate fermentation acid end products of lactobacilli or streptococci are responsible for dental caries formation [74]. Metabolomic profiles showing low levels of lactic acid in patients compared to controls could reflect the antagonistic activities between strains of lactic acid bacteria and Gram negative periodontal pathogens like *P*. *gingivalis*, *F*. *nucleatum*, and *T*. *denticola* [75]. The observed decrease of gamma-aminobutyrate (GABA) in the oral fluid of patients corresponds as well with a reduced population of LAB. Indeed, some LAB species biosynthesize GABA by glutamate decarboxylation. In addition, logistic regression showed that the combination of three metabolites (lactate, GABA, butyrate) present in proportion to their respective thresholds is associated with a high risk for periodontal disease activity (VPP of 0.77).

## Conclusion

In summary, the present study designed as a pilot study using ^1^H NMR spectroscopy analysis of saliva in periodontitis has shown its ability to identify clinically relevant biomarkers. It must be stressed that ^1^H-NMR while a fast, robust, quite simple and reproducible method for small metabolites analysis has been exceptionally tested for periodontitis on a clinical scale. It can be largely expected that, after a validation step on a larger cohort of patients, ^1^HNMR analysis of saliva may offer a considerable help in the early diagnosis and follow-up of periodontitis. The metabolomics signature obtained by ^1^H NMR suggests that the main signals identifying a periodontitis-related oral fluid milieu derive from bacterial metabolism. An alternative approach focused on the salivary microbiota in patients with periodontitis has been recently published [76]. While the method is a less easy one and completely different it would be of great interest to share the data of both methods on the same patients as dysbiosis is considered the main etiologic factor and metabolomics reflects small metabolites from the microbiota as well as from the inflammatory reaction of the host. In conclusion metabolomic profiles most probably in line with a shift in oral microbiota are a potential tool of choice for diagnosis, management, and follow-up of patients. Indeed, it needs before generalisation of the method a validation step on a larger cohort of patients and controls.

## Supporting information

S1 Table S1 Table of the univariate analysis (Mann-Whitney test) of the metabolites identitified by the OPLS loadings plot as relevant. NMR data are available on metabolight platform as EMTBLS524.
(DOCX)

## References

[1] Mombelli A. Periodontitis as an infectious disease: specific features and their implications. Oral Dis. 2003;9:6–10. doi: 10.1034/j.1601-0825.9.s1.2.x 12974524 10.1034/j.1601-0825.9.s1.2.x

[2] Hajishengallis G. The inflammophilic character of the periodontitis-associated microbiota. Mol Oral Microbiol. 2014;29(6):248–57. doi: 10.1111/omi.12065 24976068 10.1111/omi.12065 PMC4232466

[3] Trindade F, Oppenheim FG, Helmerhorst EJ, Amado F, Gomes PS, Vitorino R. Uncovering the molecular networks in periodontitis. Proteomics Clinical Applications. 2014;8(9–10):748–61. doi: 10.1002/prca.201400028 24828325 10.1002/prca.201400028 PMC4426160

[4] Nibali L. Aggressive Periodontitis: microbes and host response, who to blame? Virulence. 2015;6(3):223–8. doi: 10.4161/21505594.2014.986407 25654663 10.4161/21505594.2014.986407 PMC4601283

[5] Bourgeois D, Bouchard P, Mattout C. Epidemiology of periodontal status in dentate adults in France, 2002–2003. J Periodontal Res. 2007;42(3):219–27. doi: 10.1111/j.1600-0765.2006.00936.x 17451541 10.1111/j.1600-0765.2006.00936.x

[6] Eke PI, Dye BA, Wei L, Slade GD, Thornton-Evans GO, Borgnakke WS, et al Update on Prevalence of Periodontitis in Adults in the United States: NHANES 2009 to 2012. J Periodontol. 2015;86(5):611–22. doi: 10.1902/jop.2015.140520 25688694 10.1902/jop.2015.140520 PMC4460825

[7] Costalonga M, Herzberg MC. The oral microbiome and the immunobiology of periodontal disease and caries. Immunol Lett. 2014;162(2):22–38. doi: 10.1016/j.imlet.2014.08.017 25447398 10.1016/j.imlet.2014.08.017 PMC4346134

[8] Listgarten MA. The role of dental plaque in gingivitis and periodontitis. J Clin Periodontol. 1988;15(8):485–7. doi: 10.1111/j.1600-051X.1988.tb01019.x 3053789 10.1111/j.1600-051x.1988.tb01019.x

[9] Jiao Y, Hasegawa M, Inohara N. The Role of Oral Pathobionts in Dysbiosis during Periodontitis Development. J Dent Res. 2014;93(6):539–46. doi: 10.1177/0022034514528212 24646638 10.1177/0022034514528212 PMC4023464

[10] Lazar V, Saviuc CM, Chifiriuc MC. Periodontitis and Periodontal Disease—Innovative Strategies for Reversing the Chronic Infectious and Inflammatory Condition by Natural Products. Curr Pharm Design. 2016;22(2):230–7. doi: 10.2174/138161282202151221124307 10.2174/138161282202151221124307 26561076

[11] Sousa V, Mardas N, Farias B, Petrie A, Needleman I, Spratt D, et al A systematic review of implant outcomes in treated periodontitis patients. Clin Oral Implant Res. 2016;27(7):787–844. doi: 10.1111/clr.12684 26381260 10.1111/clr.12684

[12] Scannapieco FA, Cantos A. Oral inflammation and infection, and chronic medical diseases: implications for the elderly. Periodontol 2000. 2016;72(1):153–75. doi: 10.1111/prd.12129 27501498 10.1111/prd.12129

[13] Martin-Cabezas R, Seelam N, Petit C, Agossa K, Gaertner S, Tenenbaum H, et al Association between periodontitis and arterial hypertension: A systematic review and meta-analysis. Am Heart J. 2016;180:98–112. doi: 10.1016/j.ahj.2016.07.018 27659888 10.1016/j.ahj.2016.07.018

[14] Edgar WM. Saliva—its secretion, composition and functions. Br Dent J. 1992;172(8):305–12. 1591115 10.1038/sj.bdj.4807861

[15] Schenkels L, Veerman ECI, Amerongen AVN. Biochemical-composition of human saliva in relation to other mucosal fluids. Crit Rev Oral Biol Med. 1995;6(2):161–75. 7548622 10.1177/10454411950060020501

[16] Humphrey SP, Williamson RT. A review of saliva: Normal composition, flow, and function. J Prosthet Dent. 2001;85(2):162–9. doi: 10.1067/mpr.2001.113778 11208206 10.1067/mpr.2001.113778

[17] Segata N, Haake SK, Mannon P, Lemon KP, Waldron L, Gevers D, et al Composition of the adult digestive tract bacterial microbiome based on seven mouth surfaces, tonsils, throat and stool samples. Genome Biol. 2012;13(6). doi: 10.1186/gb-2012-13-6-r42 22698087 10.1186/gb-2012-13-6-r42 PMC3446314

[18] Estreicher A, Broggiato A, Duroux P, Andersen E, Cimasoni G. Low molecular-weight proteins in human gingival crevicular fluid. Arch Oral Biol. 1996;41(8–9):733–8. doi: 10.1016/s0003-9969(96)00076-3 9022910 10.1016/s0003-9969(96)00076-3

[19] Giannobile WV. Salivary diagnostics for periodontal diseases. J Am Dent Assoc. 2012;143(10 Suppl):6S–11S. Epub 2012/10/17. . 23024320 10.14219/jada.archive.2012.0341

[20] Range H, Leger T, Huchon C, Ciangura C, Diallo D, Poitou C, et al Salivary proteome modifications associated with periodontitis in obese patients. J Clin Periodontol. 2012;39(9):799–806. doi: 10.1111/j.1600-051X.2012.01913.x 22780105 10.1111/j.1600-051X.2012.01913.x

[21] Rosa N, Correia MJ, Arrais JP, Lopes P, Melo J, Oliveira JL, et al From the salivary proteome to the OralOme: Comprehensive molecular oral biology. Arch Oral Biol. 2012;57(7):853–64. doi: 10.1016/j.archoralbio.2011.12.010 22284344 10.1016/j.archoralbio.2011.12.010

[22] Barnes VM, Kennedy AD, Panagakos F, Devizio W, Trivedi HM, Jonsson T, et al Global Metabolomic Analysis of Human Saliva and Plasma from Healthy and Diabetic Subjects, with and without Periodontal Disease (vol 9, e105181, 2014). PLoS One. 2014;9(11). doi: 10.1371/journal.pone.0114091 10.1371/journal.pone.0105181 PMC4136819 25133529

[23] Gupta A, Govila V, Saini A. Proteomics ‚Äì The research frontier in periodontics. Journal of Oral Biology and Craniofacial Research. 2015;5(1):46–52. doi: 10.1016/j.jobcr.2015.01.001 PMC4382510. 25853048 10.1016/j.jobcr.2015.01.001 PMC4382510

[24] Trindade F, Amado F, Oliveira-Silva RP, Daniel-da-Silva AL, Ferreira R, Klein J, et al Toward the definition of a peptidome signature and protease profile in chronic periodontitis. Proteomics Clinical Applications. 2015;9(9–10):917–27. doi: 10.1002/prca.201400191 25669956 10.1002/prca.201400191

[25] Kuboniwa M, Sakanaka A, Hashino E, Bamba T, Fukusaki E, Amano A. Prediction of Periodontal Inflammation via Metabolic Profiling of Saliva. J Dent Res. 2016;95(12):1381–6. doi: 10.1177/0022034516661142 27470067 10.1177/0022034516661142

[26] Kruk J, Doskocz M, Jodlowska E, Zacharzewska A, Lakomiec J, Czaja K, et al NMR Techniques in Metabolomic Studies: A Quick Overview on Examples of Utilization. Appl Magn Reson. 2017;48(1):1–21. Epub 2017/01/24. doi: 10.1007/s00723-016-0846-9 28111499 10.1007/s00723-016-0846-9 PMC5222922

[27] de Laurentiis G, Paris D, Melck D, Maniscalco M, Marsico S, Corso G, et al Metabonomic analysis of exhaled breath condensate in adults by nuclear magnetic resonance spectroscopy. European Respiratory Journal. 2008;32(5):1175–83. doi: 10.1183/09031936.00072408 18653649 10.1183/09031936.00072408

[28] Munshi SU, Rewari BB, Bhavesh NS, Jameel S. Nuclear Magnetic Resonance Based Profiling of Biofluids Reveals Metabolic Dysregulation in HIV-Infected Persons and Those on Anti-Retroviral Therapy. PLoS One. 2013;8(5). e64298 doi: 10.1371/journal.pone.0064298 23696880 10.1371/journal.pone.0064298 PMC3655987

[29] Amathieu R, Triba MN, Goossens C, Bouchemal N, Nahon P, Savarin P, et al Nuclear magnetic resonance based metabolomics and liver diseases: Recent advances and future clinical applications. World journal of gastroenterology. 2016;22(1):417–26. Epub 2016/01/13. doi: 10.3748/wjg.v22.i1.417 26755887 10.3748/wjg.v22.i1.417 PMC4698504

[30] Yamada-Nosaka A, Fukutomi S, Uemura S, Hashida T, Fujishita M, Kobayashi Y, et al Preliminary nuclear magnetic resonance studies on human saliva. Arch Oral Biol. 1991;36(9):697–701. http://dx.doi.org/10.1016/0003-9969(91)90025-P. 1741702 10.1016/0003-9969(91)90025-p

[31] Grootveld M, Silwood CJL. H-1 NMR analysis as a diagnostic probe for human saliva. Biochem Biophys Res Commun. 2005;329(1):1–5. doi: 10.1016/j.bbrc.2005.01.112 15721264 10.1016/j.bbrc.2005.01.112

[32] Bertram HC, Eggers N, Eller N. Potential of Human Saliva for Nuclear Magnetic Resonance-Based Metabolomics and for Health-Related Biomarker Identification. Anal Chem. 2009;81(21):9188–93. doi: 10.1021/ac9020598 19780580 10.1021/ac9020598

[33] Santone C, Dinallo V, Paci M, D'Ottavio S, Barbato G, Bernardini S. Saliva metabolomics by NMR for the evaluation of sport performance. J Pharm Biomed Anal. 2014;88:441–6. doi: 10.1016/j.jpba.2013.09.021 24176749 10.1016/j.jpba.2013.09.021

[34] Duchemann B, Triba MN, Guez D, Rzeznik M, Savarin P, Nunes H, et al Nuclear magnetic resonance spectroscopic analysis of salivary metabolome in sarcoidosis. Sarcoidosis Vasc Diffus Lung Dis. 2016;33(1):10–6. 27055831

[35] Wallner-Liebniann S, Tenori L, Mazzoleni A, Dieber-Rotheneder M, Konrad M, Hofmann P, et al Individual Human Metabolic Phenotype Analyzed by H-1 NMR of Saliva Samples. J Proteome Res. 2016;15(6):1787–93. doi: 10.1021/acs.jproteome.5b01060 27087681 10.1021/acs.jproteome.5b01060

[36] Silwood CJL, Lynch E, Claxson AWD, Grootveld MC. H-1 and C-13 NMR spectroscopic analysis of human saliva. J Dent Res. 2002;81(6):422–7. doi: 10.1177/154405910208100613 12097436 10.1177/154405910208100613

[37] Aimetti M, Cacciatore S, Graziano A, Tenori L. Metabonomic analysis of saliva reveals generalized chronic periodontitis signature. Metabolomics. 2012;8(3):465–74. doi: 10.1007/s11306-011-0331-2

[38] Armitage G. Development of a classification system for periodontal diseases and conditions. Annals of Periodontology. 1999;4(1):1–6. doi: 10.1902/annals.1999.4.1.1 10863370 10.1902/annals.1999.4.1.1

[39] Grossi SG, Zambon JJ, Ho AW, Koch G, Dunford RG, Machtei EE, et al Assessment of risk for periodontal-disease .1. Risk indicators for attachment loss. J Periodontol. 1994;65(3):260–7. doi: 10.1902/jop.1994.65.3.260 8164120 10.1902/jop.1994.65.3.260

[40] Eke PI, Wei L, Borgnakke WS, Thornton-Evans G, Zhang XY, Lu H, et al Periodontitis prevalence in adults > = 65 years of age, in the USA. Periodontol 2000. 2016;72(1):76–95. doi: 10.1111/prd.12145 27501492 10.1111/prd.12145 PMC8223257

[41] Page RC, Eke PI. Case definitions for use in population—Based surveillance of periodontitis. J Periodontol. 2007;78(7):1387–99. doi: 10.1902/jop.2007.060264 17608611 10.1902/jop.2007.060264

[42] Eke PI, Page RC, Wei L, Thornton-Evans G, Genco RJ. Update of the Case Definitions for Population-Based Surveillance of Periodontitis. J Periodontol. 2012;83(12):1449–54. doi: 10.1902/jop.2012.110664 22420873 10.1902/jop.2012.110664 PMC6005373

[43] Chaves ES, Wood RC, Jones AA, Newbold DA, Manwell MA, Kornman KS. Relationship of bleeding on probing and gingival index bleeding as clinical-parameters of gingival inflammation. J Clin Periodontol. 1993;20(2):139–43. doi: 10.1111/j.1600-051X.1993.tb00328.x 8436633 10.1111/j.1600-051x.1993.tb00328.x

[44] O'Leary TJ, Drake RB, Naylor JE. The plaque control record. J Periodontol. 1972;43(1):38 Epub 1972/01/01. doi: 10.1902/jop.1972.43.1.38 4500182 10.1902/jop.1972.43.1.38

[45] Ainamo J, Bay I. Problems and proposals for recording gingivitis and plaque. Int Dent J. 1975;25(4):229–35. Epub 1975/12/01. . 1058834

[46] Lang NP, Adler R, Joss A, Nyman S. Absence of bleeding on probing—an indicator of periodontal stability. J Clin Periodontol. 1990;17(10):714–21. doi: 10.1111/j.1600-051X.1990.tb01059.x 2262585 10.1111/j.1600-051x.1990.tb01059.x

[47] Lang NP, Joss A, Orsanic T, Gusberti FA, Siegrist BE. BLEEDING ON PROBING—a predictor for the progression of periodontal-disease. J Clin Periodontol. 1986;13(6):590–6. doi: 10.1111/j.1600-051X.1986.tb00852.x 3489010 10.1111/j.1600-051x.1986.tb00852.x

[48] Hugoson A, Jordan T. Frequency-distribution of individuals aged 20–70 years according to severity of periodontal-disease. Community Dentistry and Oral Epidemiology. 1982;10(4):187–92. doi: 10.1111/j.1600-0528.1982.tb00377.x 6956481 10.1111/j.1600-0528.1982.tb00377.x

[49] Savorani F, Tomasi G, Engelsen SB. icoshift: A versatile tool for the rapid alignment of 1D NMR spectra. J Magn Reson. 2010;202(2):190–202. Epub 2009/12/17. doi: 10.1016/j.jmr.2009.11.012 . 20004603 10.1016/j.jmr.2009.11.012

[50] Dieterle F, Ross A, Schlotterbeck G, Senn H. Probabilistic quotient normalization as robust method to account for dilution of complex biological mixtures. Application in H-1 NMR metabonomics. Anal Chem. 2006;78(13):4281–90. doi: 10.1021/ac051632c 16808434 10.1021/ac051632c

[51] Kambouchner M, Levy P, Nicholson AG, Schubel K, Magois E, Feuillet S, et al Prognostic relevance of histological variants in nonspecific interstitial pneumonia. Histopathology. 2014;65(4):549–60. doi: 10.1111/his.12415 24621097 10.1111/his.12415

[52] Cuevas-Cordoba B, Santiago-Garcia J. Saliva: A Fluid of Study for OMICS. Omics-a Journal of Integrative Biology. 2014;18(2):87–97. doi: 10.1089/omi.2013.0064 24404837 10.1089/omi.2013.0064

[53] Hanioka T, Tanaka M, Takaya K, Matsumori Y, Shizukuishi S. Pocket oxygen tension in smokers and non-smokers with periodontal disease. J Periodontol. 2000;71(4):550–4. doi: 10.1902/jop.2000.71.4.550 10807117 10.1902/jop.2000.71.4.550

[54] Grant MM, Kolamunne RT, Lock FE, Matthews JB, Chapple ILC, Griffiths HR. Oxygen tension modulates the cytokine response of oral epithelium to periodontal bacteria. J Clin Periodontol. 2010;37(12):1039–48. doi: 10.1111/j.1600-051X.2010.01622.x 20955352 10.1111/j.1600-051X.2010.01622.x

[55] Almerich-Silla JM, Montiel-Company JM, Pastor S, Serrano F, Puig-Silla M, Dasi F. Oxidative Stress Parameters in Saliva and Its Association with Periodontal Disease and Types of Bacteria. Dis Markers. 2015 653537 doi: 10.1155/2015/653537 26494938 10.1155/2015/653537 PMC4606402

[56] Acquier AB, De Couto Pita AK, Busch L, Sanchez GA. Parameters of oxidative stress in saliva from patients with aggressive and chronic periodontitis. Redox Report. 2016:1–8. doi: 10.1080/13510002.2016.1198104 27320473 10.1080/13510002.2016.1198104 PMC6837673

[57] Tsai C-Y, Tang CY, Tan T-S, Chen K-H, Liao K-H, Liou M-L. Subgingival microbiota in individuals with severe chronic periodontitis. Journal of Microbiology, Immunology and Infection. 2016 http://dx.doi.org/10.1016/j.jmii.2016.04.007. 10.1016/j.jmii.2016.04.007 27262209

[58] Niederman R, Zhang J, Kashket S. Short-chain carboxylic-acid-stimulated, PMN-mediated gingival inflammation. Critical Reviews in Oral Biology & Medicine. 1997;8(3):269–90. 9260044 10.1177/10454411970080030301

[59] Uematsu H, Sato N, Hossain MZ, Ikeda T, Hoshino E. Degradation of arginine and other amino acids by butyrate-producing asaccharolytic anaerobic Gram-positive rods in periodontal pockets. Arch Oral Biol. 2003;48(6):423–9. doi: 10.1016/s0003-9969(03)00031-1 12749914 10.1016/s0003-9969(03)00031-1

[60] Chang MC, Tsai YL, Chen YW, Chan CP, Huang CF, Lan WC, et al Butyrate induces reactive oxygen species production and affects cell cycle progression in human gingival fibroblasts. J Periodontal Res. 2013;48(1):66–73. doi: 10.1111/j.1600-0765.2012.01504.x 22834967 10.1111/j.1600-0765.2012.01504.x

[61] Nishihara T, Koseki T. Microbial etiology of periodontitis. Periodontol 2000. 2004;36:14–26. doi: 10.1111/j.1600-0757.2004.03671.x 15330940 10.1111/j.1600-0757.2004.03671.x

[62] Qiqiang L, Huanxin M, Xuejun G. Longitudinal study of volatile fatty acids in the gingival crevicular fluid of patients with periodontitis before and after nonsurgical therapy. J Periodontal Res. 2012;47(6):740–9. doi: 10.1111/j.1600-0765.2012.01489.x 22594616 10.1111/j.1600-0765.2012.01489.x

[63] Socransky SS, Haffajee AD, Cugini MA, Smith C, Kent RL. Microbial complexes in subgingival plaque. J Clin Periodontol. 1998;25(2):134–44. doi: 10.1111/j.1600-051X.1998.tb02419.x 9495612 10.1111/j.1600-051x.1998.tb02419.x

[64] Klionsky DJ, Abdalla FC, Abeliovich H, Abraham RT, Acevedo-Arozena A, Adeli K, et al Guidelines for the use and interpretation of assays for monitoring autophagy. Autophagy. 2012;8(4):445–544. Epub 2012/09/12. doi: 10.4161/auto.19496 22966490 10.4161/auto.19496 PMC3404883

[65] Boris S, Barbes C. Role played by lactobacilli in controlling the population of vaginal pathogens. Microbes and Infection. 2000;2(5):543–6. doi: 10.1016/s1286-4579(00)00313-0 10865199 10.1016/s1286-4579(00)00313-0

[66] Kuramitsu HK, He XS, Lux R, Anderson MH, Shi WY. Interspecies interactions within oral microbial communities. Microbiol Mol Biol Rev. 2007;71(4):653–+. doi: 10.1128/MMBR.00024-07 18063722 10.1128/MMBR.00024-07 PMC2168648

[67] Fayol-Messaoudi D, Berger CdN, Coconnier-Polter M-Hln, Li√©vin-Le Moal V, Servin AL. pH-, Lactic Acid-, and Non-Lactic Acid-Dependent Activities of Probiotic Lactobacilli against Salmonella enterica Serovar Typhimurium. Applied and Environmental Microbiology. 2005;71(10):6008–13. doi: 10.1128/AEM.71.10.6008-6013.2005 PMC1266002. 16204515 10.1128/AEM.71.10.6008-6013.2005 PMC1266002

[68] Pessione E. Lactic acid bacteria contribution to gut microbiota complexity: lights and shadows. Front Cell Infect Microbiol. 2012;2 Unsp 86 doi: 10.3389/fcimb.2012.00086 22919677 10.3389/fcimb.2012.00086 PMC3417654

[69] Pessi T, Sutas Y, Hurme H, Isolauri E. Interleukin-10 generation in atopic children following oral Lactobacillus rhamnosus GG. Clin Exp Allergy. 2000;30(12):1804–8. doi: 10.1046/j.1365-2222.2000.00948.x 11122221 10.1046/j.1365-2222.2000.00948.x

[70] Nam H, Whang K, Lee Y. Analysis of vaginal lactic acid producing bacteria in healthy women. Journal of Microbiology. 2007;45(6):515–20. 18176534

[71] Yang RG, Johnson MC, Ray B. Novel method to extract large amounts of bacteriocins from lactic-acid bacteria. Applied and Environmental Microbiology. 1992;58(10):3355–9. 1444369 10.1128/aem.58.10.3355-3359.1992 PMC183103

[72] Alakomi HL, Skytta E, Saarela M, Mattila-Sandholm T, Latva-Kala K, Helander IM. Lactic acid permeabilizes gram-negative bacteria by disrupting the outer membrane. Applied and Environmental Microbiology. 2000;66(5):2001–5. doi: 10.1128/aem.66.5.2001-2005.2000 10788373 10.1128/aem.66.5.2001-2005.2000 PMC101446

[73] He XS, Tian Y, Guo LH, Lux R, Zusman DR, Shi WY. Oral-Derived Bacterial Flora Defends Its Domain by Recognizing and Killing Intruders-A Molecular Analysis Using Escherichia coli as a Model Intestinal Bacterium. Microb Ecol. 2010;60(3):655–64. doi: 10.1007/s00248-010-9708-4 20625713 10.1007/s00248-010-9708-4 PMC2954290

[74] Kleinberg I. A mixed-bacteriae ecological approach to understanding the role of the oral bacteria in dental caries causation: An alternative to Streptococcus mutans and the specific-plaque hypothesis. Critical Reviews in Oral Biology & Medicine. 2002;13(2):108–25. 12097354 10.1177/154411130201300202

[75] Koll-Klais P, Mandar R, Leibur E, Marcotte H, Hammarstrom L, Mikelsaar M. Oral lactobacilli in chronic periodontitis and periodontal health: species composition and antimicrobial activity. Oral Microbiol Immunol. 2005;20(6):354–61. doi: 10.1111/j.1399-302X.2005.00239.x 16238595 10.1111/j.1399-302X.2005.00239.x

[76] Kageyama S, Takeshita T, Asakawa M, Shibata Y, Takeuchi K, Yamanaka W, et al Relative abundance of total subgingival plaque-specific bacteria in salivary microbiota reflects the overall periodontal condition in patients with periodontitis. PLoS One. 2017;12(4):e0174782 Epub 2017/04/04. doi: 10.1371/journal.pone.0174782 28369125 10.1371/journal.pone.0174782 PMC5378373

