# Supplementary material for: Identification of a discriminative metabolomic fingerprint of potential clinical relevance in saliva of patients with periodontitis using 1H nuclear magnetic resonance (NMR) spectroscopy
Source: PLoS One. 2017 Aug 24;12(8):e0182767. doi: 10.1371/journal.pone.0182767 (PMC5570357; doi:10.1371/journal.pone.0182767)
Supplement: S1 Table — NMR data are available on metabolight platform as EMTBLS524. (DOCX) [file pone.0182767.s001.docx]

**Identification of a Discriminative Metabolomic Fingerprint of Potential Clinical Relevance in Saliva of Patients with Periodontitis Using^1^H Nuclear Magnetic Resonance (NMR) Spectroscopy**

***Short title: NMR Analysis of Saliva in Periodontitis***

Matthias Rzeznik^1,2¶^, Mohamed N. Triba^1¶^, Pierre Levy^3,4^, Sébastien Jungo^2^, Eliot Botosoa^1,5^, Boris Duchemann^6^, Laurence Le Moyec^7^, Jean-François Bernaudin^6,8,9^, Philippe Savarin^1*^, Dominique Guez^2*^

**Supplementary material**

| **Table S1 of the univariate analysis (Mann-Whitney test) of the metabolites identitified by the OPLS loadings plot as relevant** | |  | **n = 51** |
| --- | --- | --- | --- |
|  |  |  |  |
| **variables** | **p** |  |  |
| **Butyrate** | 0,0176 |  |  |
| 3,7_3,56 | 0,8951 |  |  |
| Tyrosine | 0,2352 |  |  |
| 3,88_3,76 | 0,4624 |  |  |
| Acetate | 0,2207 |  |  |
| 4,46_4,38 | 0,1632 |  |  |
| 4,02_3,94 | 0,0215 |  |  |
| **Hydroxybutyrate** | 0,0735 |  |  |
| 2,08_1,94 | 0,0864 |  |  |
| Uree | 0,0595 |  |  |
| **Lactate** | 0,1632 |  |  |
| **GABA** | 0,0079 |  |  |
| **Thréonine** | 0,0015 |  |  |
| Propionate | 0,8065 |  |  |

Prior to the logistic regression, a univariate analysis by a Mann –Whitney test, considering the area of the whole NMR signal for the most discriminating metabolites was used to make the selection of the metabolites that will be computed for logistic regression analysis. As 51 patients and controls were included, 5 biomarkers were selected among all the 14 identified metabolites. Variables with P ≤ 0.2 on univariate analysis and already identified in the OPLS loadings plot analysis shown in table 2 as potentially clinically relevant (selected variables in bold) were included in the stepwise regression analysis to compare patients to controls.
